# Assessing vaccine effectiveness against severe COVID-19 disease caused by omicron variant. Report from a meeting of the World Health Organization

**DOI:** 10.1016/j.vaccine.2022.04.069

**Published:** 2022-06-09

**Authors:** Daniel R. Feikin, Laith J. Abu-Raddad, Nick Andrews, Mary-Ann Davies, Melissa M. Higdon, Walter A. Orenstein, Minal K. Patel

**Affiliations:** a Department of Immunizations, Vaccines and Biologicals, World Health Organization, 20 Avenue Appia, 1211 Geneva, Switzerland; b Infectious Disease Epidemiology Group, Weill Cornell Medicine–Qatar, Cornell University, Doha, Qatar; c UK Health Security Agency, London, UK; d Health Intelligence, Western Cape Government Health, South Africa; Division of Public Health Medicine, School of Public Health and Family Medicine, Faculty of Health Sciences, University of Cape Town, South Africa; e International Vaccine Access Center, Department of International Health, Johns Hopkins Bloomberg School of Public Health, Baltimore, Maryland, USA; f Emory Vaccine Center, 1462 Clifton Road NE, Atlanta, GA, USA

**Keywords:** COVID-19, Vaccine effectiveness, Omicron variant

## Abstract

Vaccine effectiveness is lower and wanes faster against infection and symptomatic disease caused by the omicron variant of SARS-CoV-2 than was observed with previous variants. Vaccine effectiveness against severe omicron disease, on average, is higher, but has shown variability, including rapid apparent waning, in some studies. Assessing vaccine effectiveness against omicron severe disease using hospital admission as a measure of severe disease has become more challenging because of omicron’s attenuated intrinsic severity and its high prevalence of infection. Many hospital admissions likely occur among people with incidental omicron infection or among those with infection-induced exacerbation of chronic medical conditions. To address this challenge, the World Health Organization held a virtual meeting on March 15, 2022, to review evidence from several studies that assessed Covid-19 vaccine effectiveness against severe omicron disease using several outcome definitions. Data was shown from studies in South Africa, the United States, the United Kingdom and Qatar. Several approaches were proposed that better characterize vaccine protection against severe Covid-19 disease caused by the omicron variant than using hospitalization of omicron-infected persons to define severe disease. Using more specific definitions for severe respiratory Covid-19 disease, such as indicators of respiratory distress (e.g. oxygen requirement, mechanical ventilation, and ICU admission), showed higher vaccine effectiveness than against hospital admission. Second, vaccine effectiveness against progression from omicron infection to hospitalization, or severe disease, also showed higher vaccine protection. These approaches might better characterize vaccine performance against severe Covid-19 disease caused by omicron, as well as future variants that evade humoral immunity, than using hospitalization with omicron infection as an indicator of severe disease.

## 1 Background and meeting objectives

Since the emergence of the omicron variant of SARS-CoV-2 in November 2021, mounting evidence has demonstrated significant immune evasion from infection-induced and vaccine-induced immunity. Vaccine effectiveness is lower against infection and symptomatic disease caused by omicron than other variants, including delta [1]. Moreover, vaccine effectiveness against these outcomes appears to wane faster after the primary series of vaccination. Vaccine effectiveness against severe omicron disease, on average, is higher, perhaps because of the role of preserved cellular immunity [2]. Nonetheless, assessing vaccine effectiveness against omicron severe disease has become more challenging because of its attenuated intrinsic severity and its high prevalence of infection. To address this challenge, the World Health Organization held a virtual meeting of the Covid-19 Vaccine Effectiveness Methods Group to review the evidence from several studies that assessed Covid-19 vaccine effectiveness against severe omicron disease using several outcome definitions. Data was shown from studies in South Africa, the United States, the United Kingdom and Qatar. This report summarizes the results of these studies, as well as other relevant studies in the pre-print or published literature and discusses approaches to optimize evaluations of vaccine effectiveness against severe Covid-19 disease caused by omicron or future variants with immune evasion.

## 2 Vaccine effectiveness against severe disease among persons with omicron infection

Since June 2021, the World Health Organization and International Vaccine Access Center at Johns Hopkins Bloomberg School of Public Health have undertaken a living systematic review of the emerging evidence for COVID-19 vaccine effectiveness. The methods have been described elsewhere.[3], [4] Between December 3, 2021 and April 7, 2022, there were 21 vaccine effectiveness studies that met our inclusion criteria that reported results for severe omicron disease for five vaccines (Table 1 and Figure) [5], [6], [7], [8], [9], [10], [11], [12], [13], [14], [15], [16], [17], [18], [19], [20], [21], [22], [23], [24], [25], [26]. The majority (n = 13, 62%) of studies used hospitalization with some clinical evidence of Covid-19 disease as the outcome, while six (29%) studies used hospitalization with PCR-confirmed infection without clinical criteria, and 4 (19%. 4/21) used other outcomes besides hospitalization. (Two studies evaluated vaccine effectiveness for more than one severe outcome.) In contrast to vaccine effectiveness against delta severe disease, the majority of vaccine effectiveness estimates for omicron severe disease were below 75%; for example, thirteen (81%) of sixteen vaccine effectiveness estimates within three months of vaccination with the primary series were below 75% (Figure). Moreover, 13 (42%) vaccine-specific estimates fell below 50% at some point in time after vaccination. [5], [9], [14], [21], [24], [26], [17], [18], [19] Vaccine effectiveness after receipt of a booster dose increased to > 75% for all vaccines within the first 3 months after a booster dose, with the exception of one study that reported a vaccine effectiveness of 71% at 8–59 days after a homologous CoronaVac booster.[21] Few studies have evaluated vaccine effectiveness against severe omicron disease three months or more after the booster dose. There is a suggestion that the vaccine effectiveness after the primary series is lower when severe disease is defined as hospitalization without requirement for clinical criteria of Covid-19 than hospitalization with clinical criteria, particularly after 3 months since vaccination, although too few studies (n = 3) are available to make a definitive comparison (Figure).

**Table 1:** Covid-19 vaccine effectiveness against severe disease.

Study (Country)	Study Design (Variables controlled for in VE estimates)	Testing Period	Age group (years)/Study population	Severe Disease Outcome	PRIMARY SERIES			BOOSTER		
					Vaccine	Time interval since final dose (days)	Vaccine effectiveness (95% CI)	Vaccine	Time interval since booster dose (days)	Vaccine effectiveness (95% CI)
Araos (Chile)	Retrospective cohort (age, sex, geographic region, proxy for income, nationality, comorbidities	Dec 6, 2021 – Feb 26, 2022	3–5	Hospitalization with clinical criteria	Sinovac - CoronaVac	≥14	65.2 (50.4–75.6)			
				ICU admission		≥14	68.8 (18.0–88.1)			
Baum (Finland)	(age, sex, geographic region, long-term care residence, influenza vaccination, previous hospitalization, comorbidities)	Jan 1, 2022 – Feb 19, 2022	≥ 70	Hospitalization with clinical criteria (any inpatient encounter with a primary diagnosis of Covid-19, acute respiratory tract infection, or severe complications of lower respiratory tract infections)	AstraZeneca - Vaxzevria	14–90	100 (CI omitted)*	Moderna -Spikevax	14–60	100 (CI omitted)
						91–180	41 (-140–86)		≥61	40 (-336–92)*
						≥181	43 (-10–70)	Pfizer BioNTech - Comirnaty	14–60	98 (89–100)
									≥61	90 (27–99)*
					Moderna -Spikevax	14–90	92 (43–99)	Moderna -Spikevax	14–60	97 (92–99)
						91–180	90 (28–99)		≥61	92 (79–97)*
						≥181	72 (43-86)	Pfizer BioNTech - Comirnaty	14–60	96 (82–99)
									≥61	100 (CI omitted)*
					Pfizer BioNTech - Comirnaty	14–90	91 (79–96)	Pfizer BioNTech - Comirnaty	14–60	95 (94–97)
						91–180	76 (56–86)		≥61	90 (87–93)*
						≥181	61 (48–71)	Moderna -Spikevax	14–60	94 (89–97)
									≥61	48 (-13–76)*
Buchan (Canada)	Test-negative case-control (age, sex, geographic region, number of tests, prior infection, comorbidities, influenza vaccination, neighborhood median income, proportion of population employed as non-health essential workers, number persons in household, proportion of population identifying as minority)	Dec 6, 2021 – Dec 26, 2021	≥18	Hospitalization with clinical criteria (specific guidance provided to report only hospitalizations due to COVID, i.e. persons who received treatment for COVID-19)	Moderna – Spikevax or Pfizer BioNTech - Comirnaty			Pfizer BioNTech - Comirnaty	≥7	95 (87–98)
								Moderna – Spikevax	≥7	93 (74–98)
Chemaitelly (Qatar)	Test-negative case- control (matched two-to-one by sex, 10-year age group, nationality, and calendar week of PCR test)	Dec 23, 2021 – Feb 2, 2022	All	Severe, critical, or fatal disease	Moderna - Spikevax	0–179	87.1 (40.2–97.2)	Moderna - Spikevax	1–41	81.8 (-49.5–97.8)
						≥180	68.4 (46.1–81.5)		≥42	100 (CI omitted)*
					Pfizer BioNTech -Comirnaty	0–179	70.4 (45.0–84.0)	Pfizer BioNTech -Comirnaty	1–41	90.9 (78.6–96.1)
						≥180	77.5 (67.8–84.3)		≥42	90.1 (80.6–95.0)*
Collie (South Africa)	Test-negative case-control (age, sex, prior infection, calendar time, geographic region, number of CDC risk factors)	Nov 15, 2021 – Dec 7, 2021	≥18	Hospitalization	Pfizer BioNTech - Comirnaty	≥14	70 (62–76)*			
Ferdinands (USA)	Test-negative case-control (age, geographic region, calendar time, local virus circulation, propensity to be vaccinated)	Dec 16, 2021 – Jan 22, 2022	≥18	Hospitalization with clinical criteria (Hospitalization with COVID-19–like illness which includes diagnoses of acute respiratory illness such as COVID-19, respiratory failure or pneumonia, or related signs or symptoms such as cough, fever, dyspnea, vomiting, or diarrhea)	Moderna - Spikevax or Pfizer BioNTech - Comirnaty	14–59	71 (51–83)	Moderna -Spikevax or Pfizer BioNTech - Comirnaty	14–59	91 (88–93)
						60–119	65 (53–74)*		60–119	88 (85–90)*
						120–149	58 (38–71))		≥120	78 (67–85)
						≥150	54 (48–59)*			
Florentino (Brazil)	Test-negative case-control (age, sex, calendar week, geographic region, ethnicity, socioeconomic status, comorbidities, prior infection, current pregnancy or being in post-partum period)	Jan 1, 2022 – Mar 8, 2022	12–17	Hospitalization with clinical criteria (Hospitalizations reported through the SIVEP-Gripe system which reports cases of severe acute respiratory infection, which can be defined as an acute respiratory infection with onset, within the past 10 d, of fever and cough, and typically requires hospitalization.)	Pfizer BioNTech - Comirnaty	14–27	75.4 (57.3–85.9)*			
						28–41	82.1 (70.7–89.1)			
						42–55	82.8 (74.5–88.5)*			
						56–69	81.2 (73.4–86.7)*			
						70–83	83.0 (75.1–88.4)*			
						84–97	89.8 (82.1–94.2)*			
						≥98	84.9 (75.2–90.8)			
Gray (South Africa)	Test-negative case-control (age, sex, number of documented risk factors, surveillance week, period of prior infection, geographic region)	Nov 8, 2021 – Dec 17, 2021	HCW	Hospitalization	Janssen - Ad26.COV.2			Janssen - Ad26.COV.2	14–27	84 (67–92)*
									30–60	85 (54–95)
Hansen (Denmark)	Retrospective cohort (age, sex, comorbidities, geographic region, calendar time)	Dec 28, 2021 – Feb 15, 2022	≥ 12	Hospitalization (any hospital admission lasting at least 12 h and occurring no earlier than two days before, and no later than 14 days after, a positive PCR test)	Moderna - Spikevax			Moderna - Spikevax	14–30	90.2 (87.3–92.5)
									31–60	87.7 (85.3–89.7)*
									61–90	87.8 (84.5–90.4)*
									91–120	83.6 (77.7–88.0)
									≥121	77.3 (63.1–86.1)*
					Pfizer BioNTech - Comirnaty	14–30	50.5 (33.9–63.0)	Pfizer BioNTech - Comirnaty	14–30	88.8 (87.3–90.1)
						31–60	48.5 (36.6–58.2)*		31–60	88.5 (87.4–89.6)*
						61–90	42.6 (26.9–54.9)*		61–90	84.9 (83.1–86.5)*
						91–120	47.2 (33.7–57.9)		91–120	79.0 (76.5–81.3)
						≥121	51.6 (47.2–55.6)*		≥121	66.2 (61.1–70.7)*
Lauring (USA)	Test-negative case-control (age, sex, geographic region, calendar time, race/ethnicity)	Dec 26, 2021 – Jan 14, 2022	≥18	Hospitalization with clinical criteria (Hospitalization with a clinical syndrome consistent with acute covid-19: ≥1 of fever, cough, shortness of breath, loss of taste, loss of smell, use of respiratory support for the acute illness, or new pulmonary findings on chest imaging consistent with pneumonia)	Moderna – Spikevax or Pfizer BioNTech - Comirnaty	≥14	65 (51–75)	Moderna – Spikevax or Pfizer BioNTech - Comirnaty	7+	86 (77–91)
Natarajan (USA)	Test-negative case-control (age, calendar week, geographic region, local virus circulation, comorbidities, propensity to be vaccinated)	Dec 16, 2021 – Mar 7, 2022	≥18	Hospitalization with clinical criteria (Hospitalization with COVID-19–like illness which includes diagnoses of acute respiratory illness such as COVID-19, respiratory failure or pneumonia, or related signs or symptoms such as cough, fever, dyspnea, vomiting, or diarrhea)	Janssen-Ad26.COV2.S	≥14	31 (21–40)*	Janssen-Ad26.COV2.	7+	67 (52–77)*
								Moderna - Spikevax or Pfizer BioNTech - Comirnaty	7+	78 (70–84)*
					Moderna -Spikevax or Pfizer BioNTech - Comirnaty			Moderna - Spikevax or Pfizer BioNTech - Comirnaty	7+	90 (88–91)*
Price (USA)	Test-negative case-control (age, sex, calendar time, geographic region, race, ethnicity)	Dec 19, 2021 – Feb 17, 2022	5–11	Hospitalization with clinical criteria (Hospitalization with COVID 19 as primary reason for admission or with a clinical syndrome consistent with acute COVID-19: one or more fever, cough, shortness of breath, loss of taste, loss of smell, gastrointestinal symptoms, receipt of respiratory support, or new pulmonary findings on chest imaging.) Hospitalization	Pfizer BioNTech - Comirnaty	≥14	68 (42–82)			
			12–18		Pfizer BioNTech - Comirnaty	14–160	43 (-1–68)			
						161–314	38 (-3–62)			
Ranzani (Brazil)	Test-negative case-control (age, comorbidities, race, prior symptomatic infection)	Dec 25, 2021 – Mar 10, 2022	≥18	Hospitalization with clinical criteria (Hospitalizations reported through the SIVEP-Gripe system which reports cases of severe acute respiratory infection, which can be defined as an acute respiratory infection with onset, within the past 10 d, of fever and cough, and typically requires hospitalization.)	Sinovac- Coronavac	14–59	49.9 (30.7–63.7)	Sinovac- Coronavac	8–59	71.3 (60.3–73.2)
						60–179	62.6 (58.5–66.3)		≥60	65.4 (61.5–68.8)
						≥180	57 (53.5–60.2)	Pfizer BioNTech - Comirnaty	8–59	85.5 (83.8–87.0)
									≥60	86.1 (85.0–87.1)
Šmíd (Czech Republic)	Retrospective cohort (age group, sex and prior infection)	Dec 7, 2021 – Feb 13, 2022	≥5	Hospitalization (hospital admission of a person, who tested positive on a PCR test, within two weeks after the confirmed infection or earlier)	AstraZeneca - Vaxzevria	75–134	−139 (-861–41)			
						≥135	13 (-8–30)			
					Janssen - Ad26.COV2.S	14–74	28 (–22–57)			
						75–134	40 (-8–66)			
						≥135	38 (8–58)			
					Moderna - Spikevax	14–74	51 (-20–80)	Moderna - Spikevax	14–74	89 (84–93)
						75–134	39 (-92–81)		≥75	84 (72–91)*
						≥135	31 (9–49)			
					Pfizer BioNTech -Comirnaty	14–74	46 (28–60)	Pfizer BioNTech -Comirnaty	14–74	86 (84–89)
						75–134	−10 (-51–19)		≥75	79 (74–82)*
						≥135	34 (24–42)			
Stowe (UK)	Test-negative case-control (age, sex, index of multiple deprivation, calendar week, health and social care worker status, clinical risk group, clinically extremely vulnerable, severely immunosuppressed, prior infection)	Nov 22, 2021 – Feb 2, 2022	18–64	Hospitalization with clinical criteria (Hospitalization for at least 2 days stay and ARI code in primary diagnostic field)	AstraZeneca - Vaxzevria	14–174	59 (31.9–75.3)	Moderna - Spikevax	7–13	97.2 (86.1–99.4)*
									14–34	93.0 (86.4–96.4)
									35–69	89.2 (82.5–93.3)*
								Pfizer BioNTech -Comirnaty	7–13	90.2 (78.1–95.6)*
						≥175	53 (41.7–62)		14–34	88.9 (83.8–92.4)
									35–69	83.9(79.1–87.5)*
									≥70	82.2(76.3–86.7)*
									≥105	69 (50.3–80.7)
					Pfizer BioNTech -Comirnaty	14–174	73.8 (62.5–81.7)	Pfizer BioNTech -Comirnaty	7–13	85.2 (47.1–95.8)*
									14–34	79.7 (66.3–87.7)
									35–69	86.6 (81.3–90.4)*
						≥175	65.1 (51.3–74.9)		≥70	79.3 (71.3–85.0)*
									≥105	66.0 (44.5–79.2)
								Moderna - Spikevax	14–34	94.3 (85.0–97.8)
									35–69	89.8 (77.9–95.3)*
			≥65		AstraZeneca - Vaxzevria	14–174	71.2 (50–83.4)	Moderna - Spikevax	14–34	92.9 (87.7–95.9)*
									35–69	92.7 (89.1–95.2)
									≥70	91.8 (85.9–95.3)
								Pfizer BioNTech -Comirnaty	7–13	85.4 (73.4–92.0)*
						≥175	53.1 (43.4–61.2)		14–34	91.3 (88.5–93.5)
									35–69	89.2 (87.1–91.0)*
									≥70	87.6 (85.2–89.6)*
									≥105	86.1 (82.5–88.9)
					Pfizer BioNTech -Comirnaty	14–174	87.6 (79.4–92.5)	Pfizer BioNTech -Comirnaty	7–13	86.4 (69.1–94.0)*
									14–34	90.0 (85.4–93.2)
									35–69	88.4 (85.7–90.6)*
									≥70	88.4 (86.2–90.2)*
									≥105	85.2 (82.1–87.7)
						≥175	65.4 (56.6–72.5)	Moderna - Spikevax	7–13	92.9 (50.2–99.0)*
									14–34	92.9 (83.0–97.1)
									35–69	90.9 (84.8–94.5)*
									≥70	97.3 (90.8–99.2)
Tartof (USA)	Test-negative case- control (age, sex, race/ethnicity, body mass index, Charlson comorbidity index, prior infection)	Dec 1, 2021 – Jan 11, 2022	≥ 18	Hospitalization with clinical criteria (Hospitalization for covid-like illness: with 1 or more COVID-19 symptoms)	Pfizer BioNTech -Comirnaty	7–89	70 (41–84)	Pfizer BioNTech -Comirnaty	14–89	89 (83–92)
						90–179	67 (44–80)		≥90	90 (57–98)*
						≥180	68 (56–76)			
Tenforde (USA)	Case-control (age, sex, geographic region, calendar time, race and ethnicity)	Dec 26, 2021 – Jan 24, 2022	≥18	Invasive mechanical ventilation or in-hospital death	Moderna – Spikevax or Pfizer BioNTech - Comirnaty	≥14	79 (66–87)*	Moderna – Spikevax or Pfizer BioNTech - Comirnaty	≥7	94 (88–97)*
Thompson (USA)	Test-negative case-control (age, geographic region, calendar time, local virus circulation, propensity to be vaccinated)	Dec 16, 2021 – Jan 5, 2022	≥18	Hospitalization with clinical criteria (Hospitalization with COVID-19–like illness which includes diagnoses of acute respiratory illness such as COVID-19, respiratory failure or pneumonia, or related signs or symptoms such as cough, fever, dyspnea, vomiting, or diarrhea)	Moderna – Spikevax or Pfizer BioNTech - Comirnaty	14–179	81 (65–90)	Moderna – Spikevax or Pfizer BioNTech - Comirnaty	≥14	90 (80–94)*
						≥180	57 (39–70)			
Tseng (USA)	Test-negative case- control (age group, sex, race/ethnicity, comorbidities, frailty index, prior infection, number of healthcare encounters, specimen type, medical center area)	Dec 6, 2021 – Dec 31 2021	≥ 18	Hospitalization with clinical criteria (Hspitalization with a SARS-CoV-2-positive test or hospitalization ≤ 7 days after a SARS-CoV-2-positive test. COVID-19 hospitalization was confirmed by manual chart review conducted by a physician investigator (B.K.A.) to verify the presence of severe COVID-19 symptoms)	Moderna - Spikevax	≥14	84.5 (23.0–96.9)*	Moderna - Spikevax	≥14	99.2 (76.3–100.0)
UKHSA/ Andrews (UK)	Test-negative case- control (age group, sex, index of multiple deprivations (quintile), ethnic group, geographic region, period (day of test), health and social care worker status, clinical risk group status, clinically extremely vulnerable, and previously testing positive)	Nov 27, 2021 -Jan 23, 2022.	≥ 18	Hospitalization	AstraZeneca - Vaxzevria	140–174	55.8 (34.1–70.3)	Moderna - Spikevax	14–34	91.4 (86.8–94.4)
									35–69	91.2 (82.8–95.5)*
								Pfizer BioNTech - Comirnaty	14–34	86.9 (82.8–90.1)
						≥175	32.7 (19.7–43.6)		35–69	85 (81.2–88)*
									70–104	77.5 (69.9–83.3)
					Pfizer BioNTech - Comirnaty	14–34	73.6 (40.7–88.3)	Pfizer BioNTech - Comirnaty	14–34	88.2 (82.7–91.9)
						35–69	71.7 (49.4–84.1)*		35–69	84.5 (80.5–87.7)*
									70–104	75.8 (69.7–80.6)
						70–104	53.9 (35.3–67.1)*			
								Moderna - Spikevax	14–34	92.0 (83.0–96.2)
						105–139	59.9 (48.4–68.8)			
						140–174	57.3 (42.7–68.2)*		35–69	93.7 (80.3–98.0)*
						≥175	34.9 (17.7–48.4)			
Young-Xu (USA)	Test-negative case-control (age, sex, geographic region, comorbidities,	Dec 1, 2021 – Jan 14, 2022	Veterans ≥ 18	Hospitalization	Moderna – Spikevax or Pfizer BioNTech - Comirnaty	≥14	44 (26–58)*	Moderna – Spikevax or Pfizer BioNTech - Comirnaty	≥14	87 (80–91)*
				Death		≥14	75 (52–87)*		≥14	94 (85–98)*

Abbreviations: HCW, healthcare workers. * Not included in plot for one of following reasons: another similar time interval was available, time interval is large and cannot be placed into a specific time period for plot, or VE estimate not reliable (may apply to VE of 100% where small numbers preclude informative confidence intervals).

**Figure:**
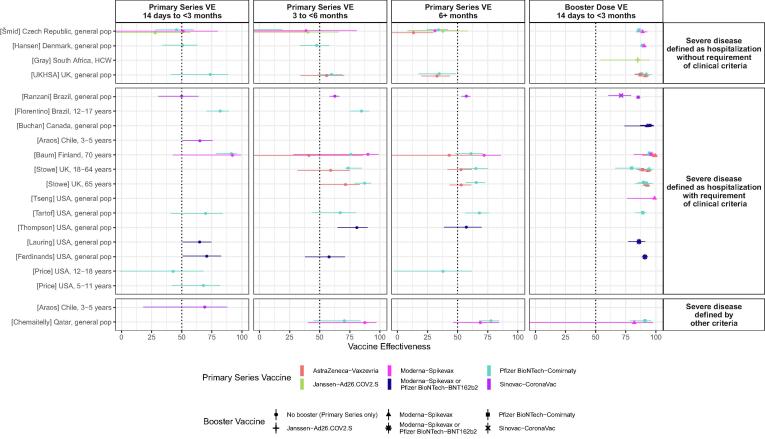

## 3 Hospitalization *with* omicron infection rather than *for* omicron disease

Hospitalization is an accessible and easily defined measure of severe disease, particularly when using electronic databases. However, criteria for hospitalization vary significantly by geographic location, individual hospital or even the stage of a Covid-19 wave, where factors like standard of care, reimbursement structure, and existing bed capacity can affect thresholds for hospital admission. Particularly in the setting of Covid-19 disease, hospitalization policies might have changed considerably. For example, in Hong Kong, prior to February 16, every person testing positive for SARS-CoV-2, regardless of their clinical status, was hospitalized.[27] For these reasons, guidance from WHO on evaluating Covid-19 vaccine effectiveness recommends that, in addition to hospital admission, severe disease definitions should also include clinical criteria that could better align results across settings.[28].

Despite these concerns, hospitalization seemed to be a fairly accurate surrogate for severe pre-omicron Covid-19 disease, showing consistently high vaccine effectiveness estimates.[29] With omicron, however, hospitalization might be a less accurate predictor of severe Covid-19 disease. First, SARS-CoV-2 infection can trigger an exacerbation of underlying medical conditions, such as chronic lung or heart disease, as occurs with other respiratory viruses, such as influenza and respiratory syncytial virus.[30], [31], [32] Second, SARS-CoV-2 infection can occur incidentally among persons hospitalized for non-Covid-19 illnesses, where SARS-CoV-2 infection is not in the causal chain leading to admission. With the large omicron wave, which was typically larger than all pre-omicron waves, but with reduced severity, the likelihood of COVID-19 diagnosis coincidental with hospital admission increased. Many hospitals test all admitted persons for SARS-CoV-2 as part of infection control protocols, yet administrative coding may not differentiate those persons admitted *with* SARS-CoV-2 infection from those admitted *for* Covid-19 disease.

An example of the changing distribution of types of Covid-19 hospitalization with omicron was presented from Western Cape Province, South Africa.[33] A detailed assessment of deaths among persons admitted with SARS-CoV-2 infection found that the percentage of deaths due to severe COVID-19 decreased from 78% during the delta wave to 50% during the omicron wave. Conversely, Covid-19 associated deaths (where SARS-CoV-2 infection may have played a role in exacerbation of underlying illnesses) and incidental infection increased from 2% and 0%, respectively, during the delta wave to 24% and 6%, respectively, during the omicron wave. Others studies have shown similar findings. In a California hospital, 19.8% of admissions with omicron infection were deemed be not likely due to Covid-19; the median age of these admissions was 38 years old compared to 67 years old for those admitted likely due to Covid-19 [34]. In one large medical center in the Netherlands, medical records review of all admissions with omicron infection during a two month period revealed that 45% were admitted for primary Covid-19 disease, 21% due to omicron infection contributing to an underlying illness, 31% due to incidental omicron infection, and 3% with an indeterminant role of omicron infection [35].

## 4 Approaches to evaluating vaccine effectiveness of severe COVID-19 disease due to omicron

At the meeting, data were presented from studies that evaluated vaccine effectiveness using other approaches to define vaccine effectiveness against severe omicron disease besides hospitalization. First, outcomes that reflect greater severity than hospital admission, particularly those more specific for hypoxic respiratory disease, such as use of high-flow oxygen, mechanical ventilation and admission to the intensive care unit, likely better assess the protection of vaccines against severe Covid-19 disease. An analysis from the United Kingdom was presented that showed that the more specific the case definition was for respiratory disease (i.e., primary ICD-10 code for respiratory illness) and severe disease (i.e., oxygen use, mechanical ventilation or ICU admission) caused by omicron variant, the higher the vaccine effectiveness [23]. For example, among SARS-CoV-2-positive 18–64 year old persons admitted for at least one day who did not have respiratory disease as their primary diagnosis the vaccine effectiveness at 14–174 days after vaccination with an mRNA vaccine or AstraZeneca-Vaxzevria was 29.5% (15.1 to 41.5), which increased to 79.1% (-36.9 to 96.8) when the admission was two or more days, had acute respiratory illness in the primary diagnosis, and required supplemental oxygen. This difference in vaccine effectiveness among hospitalized cases based on case definition was of greater magnitude with omicron than delta. Moreover, waning of the effectiveness against “severe” omicron disease over time was substantial using all admissions, but was much less when using more specific definitions for severe Covid-19 disease; whereas, with severe delta disease minimal waning of vaccine effectiveness was observed using all case definitions of severity, including hospital admission.

Two studies from the United States showed similar differences in the vaccine effectiveness for severe omicron disease based on the definition used. One study presented from the IVY network of 21 hospitals in the United States showed that vaccine effectiveness against two doses of mRNA vaccines for hospital admission with omicron was 65% (95% CI, 51–75%), while it was 79% (95% CI, 66–87%) for invasive mechanical ventilation or in-hospital death [15], [16]. The difference in vaccine effectiveness against these same outcomes was less during the delta period for two mRNA doses – 88% (95% CI 86–90%) and 85% (95% CI, 83–87%), respectively. In general, omicron patients were older and more medically complex than delta patients, suggesting a higher likelihood of exacerbation of comorbid conditions. A study that became available as a preprint subsequent to the meeting found that among hospitalized adolescents 12–18 years of age in the U.S. the vaccine effectiveness against hospitalization during the omicron-predominant-period was 40% (95% CI 9–60%) for the Pfizer-BioNTech-Comirnaty vaccine, but 79% (95% CI 51–91%) for critical Covid-19 disease (i.e., requiring life support or progressing to death); in contrast, there was minimal difference during the delta-predominant-period – 92% (95% CI 89–95%) and 96% (95% CI 90–98%), respectively.[26].

A second approach is assessing the effectiveness against progression to severe disease conditional upon being infected. Halloran et. al. conceptualized vaccine effectiveness against a disease outcome as a product of vaccine effectiveness against susceptibility to infection (i.e., VE_s_) and vaccine effectiveness against progression from infection to the disease outcome (i.e., VE_p_) [36]. As such, if VE_s_ for omicron infection decreases, the effectiveness against severe omicron disease would also apparently decrease, even if VE_p_ from infection to severe disease was maintained, thereby obscuring the component of effectiveness that prevents progression to severe disease (VE_p_). For example if VE_s_ reduces from 80% to 50%, but VE_p_ is maintained at 70%, then the overall effectiveness against hospitalization, which is 1-(1-VE_s_)*(1- VE_p_), reduces from 1-(1–0.8)*(1–0.7) = 94% to 1-(1–0.5)*(1–0.7) = 85%.

Data was presented from an unpublished analysis from Qatar using multivariable logistic regression to assess associations with progression to COVID-19 hospitalization and death among infected cases. (Supplement S1 for methods). In this setting of a young population, two or three doses of either mRNA vaccine reduced hospital admission among infected persons by 25% (95% CI, 19–31%) and 31% (95% CI 21–40%), respectively (Table 2, noting that VE_p_ = 1 – adjusted odds ratio). In contrast, vaccine protection against progression to ICU admission, mechanical ventilation or death increased to 67% (54–76%) and 84% (71–91%) for two or three doses, respectively. Additionally, the analysis showed that vaccine protection against progression from infection to severe outcomes was significantly higher when using WHO disease classifications based on clinical criteria of severity (i.e., severe Covid-19, critical Covid-19 and fatal Covid-19), than when using hospital admission (Table 2).

**Table 2:** Multivariable logistic regression investigating associations with COVID-19 hospitalization and death among persons with omicron infection. Qatar, December 19, 2021 to February 6, 2022.

Predictors	COVID-19 severity based on hospital admission criteria				COVID-19 severity based on WHO classification for infection severity					
	Any hospital admission with COVID-19* vs. mild/asymptomatic infection		ICU/mechanical ventilation/death with COVID-19 vs. mild/asymptomatic infection		Severe‡ COVID-19 vs. mild/asymptomatic infection		Critical‡ COVID-19 vs. mild/asymptomatic infection		Fatal‡ COVID-19 vs. mild/asymptomatic infection	
	aOR (95% CI)	P-value	aOR (95% CI)	P-value	aOR (95% CI)	P-value	aOR (95% CI)	P-value	aOR (95% CI)	P-value
**Vaccination status**§										
Unvaccinated	Reference		Reference		Reference		Reference		Reference	
One dose	1.66 (1.23–2.23)	0.001	0.90 (0.22–3.75)	0.883	0.83 (0.20–3.51)	0.801	1.74 (0.22–13.5)	0.597	2.13 (0.26–17.13)	0.478
Two doses	0.75 (0.69–0.81)	<0.001	0.33 (0.24–0.46)	<0.001	0.27 (0.19–0.37)	<0.001	0.13 (0.06–0.30)	<0.001	0.12 (0.05–0.28)	<0.001
Three doses	0.69 (0.60–0.79)	<0.001	0.16 (0.09–0.29)	<0.001	0.12 (0.06–0.21)	<0.001	0.07 (0.02–0.34)	0.001	0.03 (0.00–0.25)	0.001
**Prior infection**¶										
No	Reference		Reference		Reference		Reference		NA	NA
Yes	0.93 (0.82–1.04)	0.287	0.69 (0.38–1.24)	0.206	0.24 (0.09–0.65)	0.005	0.31 (0.04–2.26)	0.246	NA	NA
**Age (years)**										
0–5	Reference		Reference		Reference		Reference		Reference	
6–11	0.21 (0.16–0.29)	<0.001	0.41 (0.15–1.10)	0.077	NA	NA	NA	NA	NA	NA
12–17	0.40 (0.31–0.53)	<0.001	0.19 (0.04–0.90)	0.036	0.32 (0.03–3.10)	0.326	1.05 (0.15–7.61)	0.959	NA	NA
18–29	0.85 (0.69–1.04)	0.113	0.29 (0.12–0.72)	0.008	0.51 (0.12–2.15)	0.355	0.23 (0.02–2.70)	0.245	NA	NA
30–39	0.81 (0.66–0.99)	0.038	0.38 (0.17–0.87)	0.023	0.62 (0.16–2.38)	0.483	0.37 (0.05–2.85)	0.341	NA	NA
40–49	0.76 (0.62–0.94)	0.010	0.65 (0.29–1.49)	0.309	1.72 (0.48–6.11)	0.402	NA	NA	0.49 (0.03–8.43)	0.621
50–59	0.88 (0.71–1.09)	0.228	1.09 (0.48–2.46)	0.84	2.85 (0.81–9.96)	0.101	1.01 (0.16–6.51)	0.995	0.95 (0.07–12.81)	0.967
≥60	1.80 (1.45–2.24)	<0.001	4.67 (2.15–10.15)	<0.001	17.43 (5.22–58.25)	<0.001	3.36 (0.57–19.92)	0.183	9.56 (0.93–98.06)	0.057
**Sex**										
Female	Reference		Reference		Reference		Reference		Reference	
Male	0.72 (0.67–0.77)	<0.001	2.01 (1.47–2.74)	<0.001	1.96 (1.42–2.71)	<0.001	1.75 (0.84–3.66)	0.135	1.83 (0.85–3.92)	0.123
**Nationality**										
Qatari	Reference		Reference		Reference		Reference		Reference	
CMW nationalities**	0.78 (0.71–0.85)	<0.001	0.72 (0.49–1.08)	0.11	0.52 (0.32–0.85)	0.009	0.47 (0.15–1.49)	0.199	NA	NA
Other nationalities	0.87 (0.80–0.94)	0.001	0.67 (0.47–0.96)	0.031	0.82 (0.57–1.18)	0.291	0.45 (0.17–1.16)	0.097	0.81 (0.34–1.91)	0.63
**Comorbidity count**										
None	Reference		Reference		Reference		Reference		Reference	
1–2	2.35 (2.12–2.60)	<0.001	1.39 (0.77–2.53)	0.275	1.95 (0.99–3.82)	0.052	4.83 (1.27–18.36)	0.021	NA	NA
≥3	3.46 (3.11–3.85)	<0.001	5.90 (3.84–9.07)	<0.001	6.64 (4.14–10.65)	<0.001	15.39 (4.08–58.07)	<0.001	9.26 (2.43–35.33)	0.001

Abbreviations: aOR, adjusted odds ratio; CI, confidence interval; CMW, craft and manual workers; COVID-19, coronavirus disease 2019; ICU, intensive care unit; NA, not applicable; WHO, World Health Organization.

**These include Indians, Pakistanis, Bangladeshis, Nepalese, Sri Lankan, and Sudanese due to large proportions of these nationals being craft and manual workers.

^†^ICU/mechanical ventilation/death refers to hospitalization with COVID-19 that required ICU admission or mechanical ventilation, or that resulted in death.

* Hospital admission with COVID-19 refers to any severe acute respiratory syndrome coronavirus 2 (SARS-CoV-2) infection that was associated with hospitalization.

** These include Indians, Pakistanis, Bangladeshis, Nepalese, Sri Lankan, and Sudanese due to large proportions of these nationals being craft and manual workers.

‡ Severity,[7] criticality,[7] and fatality [8] were defined according to the WHO guidelines.

§ Vaccination status was ascertained at time of infection diagnosis.

¶ Prior infection status refers to any record of a PCR-positive or rapid-antigen-positive test ≥ 90 days before the study test.

In the IVY network in the United States, the overall vaccine effectiveness for two or three doses of mRNA vaccines among immunocompetent adults was 44% (95% CI 0–69) against progression among persons admitted with omicron infection to invasive mechanical ventilation or death, similar to what they found for delta variant (50%, 95% CI 37–60%) [16]. In the Western Cape Province, South Africa, protection of the primary series of Janssen-Ad26.COV2.S or Pfizer-BioNTech-Comirnaty vaccines against progression from infection to severe admission or death was similar during the omicron wave (adjusted HR 0.45, 95% confidence intervals, 0.36–0.56) as during the delta wave (adjusted HR 0.53, 95% confidence intervals, 0.44–0.64) [37].

In a study among members of Kaiser Permanente Southern California (not presented at meeting), the primary series of the Janssen-Ad26.COV2.S and both mRNA vaccines both showed approximately a halving of the probability of progression from omicron infection diagnosed in the outpatient setting to hospital admission (hazards ratio for progression to admission for Janssen-Ad26.COV2.S of 0.51, 95% CI 0.33–0.78, and for mRNA vaccines given ≤ 90 days prior to testing of 0.49, 95% CI 0.32–0.76.)[38] Vaccine protection against progression of omicron infection was similar to that found for delta for Ad26.COV2.S (HR 0.46, 95% CI 0.30–0.70), although vaccine protection was less with omicron than delta with the mRNA vaccines (HR 0.28, 95% CI 0.23–0.33). Minimal waning was seen in protection against progression of the mRNA vaccines with time since vaccination.

## 5 Evaluating vaccine effectiveness against omicron-associated fatality

Most studies showed high vaccine effectiveness against omicron-associated death. In Qatar, the adjusted odds ratio of progression from omicron infection to death was 0.12 (95% CI, 0.05–0.28) for two doses and 0.03 (95% CI, 0.00–0.25) for three doses of mRNA vaccines (Table 2). In South Africa, the hazard ratio of progression from omicron infection to death during the omicron wave was 0.24 (95% CI, 0.10–0.58).[37] In a study (not presented at the meeting), among U.S. veterans, two doses of the mRNA vaccines had a vaccine effectiveness of 44% (26–58) against hospitalization with omicron, compared with 75% (52–87%) against death with omicron.[5] Despite these findings, potential concern was raised in using death as an outcome for vaccine effectiveness evaluations. Death among persons who have tested positive for SARS-CoV-2 is clearly a more severe outcome than hospitalization, however, it might also be non-specific for Covid-19, particularly during the omicron wave with high infection rates. This can occur because most definitions of Covid-19-associated deaths include a positive test up to a month prior to death. Misclassification of the cause of death might be a particular concern among elderly persons with comorbidities who are at higher risk of dying from other causes. When using death as an outcome, verification of the cause of death as due to Covid-19 should be done, if feasible.

## 6 Summary and conclusions

Covid-19 vaccines likely have higher effectiveness against severe omicron Covid-19 disease than indicated by effectiveness estimates that use hospital admission of omicron-infected persons to define severe disease. This is because a greater proportion of admissions are associated with, but not caused by, omicron infection, against which current Covid-19 vaccines are less effective. To evaluate vaccine protection against severe omicron disease, we recommend using more specific definitions for severe Covid-19 respiratory disease among hospitalized persons. As a second approach to measuring vaccine protection against severe disease, we suggest evaluating progression from omicron infection to more severe outcomes, like intensive care using admission and ventilatory support. While fatal outcomes can be used to evaluate vaccine effectiveness against severe omicron disease, caution should be taken to prevent misclassification of the cause of death. It may also be useful to use ecological analyses on end points not dependent on testing, such as all cause deaths or all respiratory deaths/hospitalizations /ICU admissions, as a sense check, because in the context of high infection, it would be surprising to see these indicators remaining at low levels (as has been the case in many countries) if vaccine effectiveness against these end points was not high. Which type of severe outcomes are prevented by Covid-19 vaccines has implications for vaccine policy. Preserved high vaccine effectiveness against severe Covid-19 disease attributed to omicron suggests that the current vaccine formulations continue to have utility in preventing the most severe forms of disease. However, because omicron evades vaccine-induced immunity against infection, as perhaps will future emergent variants, a greater proportion of hospitalizations and deaths may be caused by infection-associated exacerbations of chronic diseases in vulnerable adults. Preventing these types of severe outcomes related to SARS-CoV-2 infection might require more frequent boosters or new vaccines that more effectively and durably prevent SARS-CoV-2 infection.

## Declaration of Competing Interest

Dr. Walter Orenstein is an uncompensated member of the Scientific Advisory Board for Moderna.
