# Supplementary material for: Assessing vaccine effectiveness against severe COVID-19 disease caused by omicron variant. Report from a meeting of the World Health Organization
Source: Vaccine. 2022 Jun 9;40(26):3516–27. doi: 10.1016/j.vaccine.2022.04.069 (PMC9058052; doi:10.1016/j.vaccine.2022.04.069)
Supplement: Supplementary data 1 [file mmc1.docx]

**Assessing vaccine effectiveness against severe COVID-19 disease caused by omicron variant. Report from a meeting of the World Health Organization**

**Supplemental materials**.

**S1. Methods for analysis of vaccine protection against severe disease in Qatar**

The study was conducted in the resident population of Qatar. Data sources were the national, federated databases for COVID-19 hospitalization, death, laboratory testing, and vaccination, retrieved from the integrated nationwide digital-health information platform. These databases include all SARS-CoV-2 and COVID-19 related data, and associated demographic information, with no missing information, since pandemic onset.

Two different criteria were used to determine infection severity. The first one is based on hospital admission with COVID-19 and includes two severe outcomes: acute-care-bed hospital admission with COVID-19 and ICU/mechanical ventilation/death with COVID-19.

The second criteria is based on the World Health Organization (WHO) guidelines for classification of COVID-19 case severity into three outcomes: severe COVID-19,^7^ critical COVID-19,^7^ and fatal COVID-19.^8^ This is a specific criteria in that each hospital admission with COVID-19 is investigated to determine the severity of the SARS-CoV-2 infection. Given the high specificity, some of the hospital admissions with COVID-19 may not be classified under severe COVID-19,^7^ critical COVID-19,^7^ or fatal COVID-19,^8^ as they do not fulfill the case definition for any of these three outcomes.

The WHO-classification infection severity assessment was conducted by trained medical personnel using individual chart reviews, for every patient hospitalized with COVID-19, every three days until discharge or death. Severe COVID-19 disease was defined per WHO classification as a SARS-CoV-2 infected person with “oxygen saturation of <90% on room air, and/or respiratory rate of >30 breaths/minute in adults and children >5 years old (or ≥60 breaths/minute in children <2 months old or ≥50 breaths/minute in children 2-11 months old or ≥40 breaths/minute in children 1–5 years old), and/or signs of severe respiratory distress (accessory muscle use and inability to complete full sentences, and, in children, very severe chest wall indrawing, grunting, central cyanosis, or presence of any other general danger signs)”.^9^ Detailed WHO criteria for classifying SARS-CoV-2 infection severity can be found in the WHO technical report.^9^ Critical COVID-19 disease was defined per WHO classification as a SARS-CoV-2 infected person with “acute respiratory distress syndrome, sepsis, septic shock, or other conditions that would normally require the provision of life sustaining therapies such as mechanical ventilation (invasive or non-invasive) or vasopressor therapy”.^9^ Detailed WHO criteria for classifying SARS-CoV-2 infection criticality can be found in the WHO technical report.^9^ COVID-19 death was defined per WHO classification as “a death resulting from a clinically compatible illness, in a probable or confirmed COVID-19 case, unless there is a clear alternative cause of death that cannot be related to COVID-19 disease (e.g. trauma). There should be no period of complete recovery from COVID-19 between illness and death. A death due to COVID-19 may not be attributed to another disease (e.g. cancer) and should be counted independently of preexisting conditions that are suspected of triggering a severe course of COVID-19”. Detailed WHO criteria for classifying COVID-19 death can be found in the WHO technical report.^8^

Individuals who progressed to severe, critical, or fatal COVID-19 between the time of the PCR-positive or rapid-antigen-positive test and the end of the study were classified based on their worst disease outcome, starting with death,^8^ followed by critical disease,^7^ and then severe disease.^7^

**Statistical methods**

Potential predictors of COVID-19 hospitalization and death were specified a priori and included vaccination status at the time of infection diagnosis, prior infection status (any record of a PCR-positive or rapid-antigen-positive test ≥90 days before the study test), age group, sex, nationality group, and co-morbidity count.

Multivariable logistic regression models factoring all predictors were implemented to evaluate associations with COVID-19 hospitalization and death. Adjusted odds ratios (aORs), 95% confidence intervals (CIs), and p-values were reported. P-value <0.05 indicated statistical significance. All analyses were implemented using IBM-SPSS version 27.0 (Armonk, NY, USA). The analyses were based on infections diagnosed between December 19, 2021 and February 6, 2022, that is during the Omicron wave in Qatar.

**References for supplement**

1. World Health Organization. Tracking SARS-CoV-2 variants. Available from: <https://www.who.int/en/activities/tracking-SARS-CoV-2-variants/>. 2021.

2. Qatar viral genome sequencing data. Data on randomly collected samples. <https://www.gisaid.org/phylodynamics/global/nextstrain/>. 2021. at <https://www.gisaid.org/phylodynamics/global/nextstrain/>.)

3. Altarawneh HN, Chemaitelly H, Hasan MR, et al. Protection against the Omicron Variant from Previous SARS-CoV-2 Infection. N Engl J Med 2022.

4. Chemaitelly H, Ayoub HH, Coyle P, et al. Protection of Omicron sub-lineage infection against reinfection with another Omicron sub-lineage. medRxiv 2022:2022.02.24.22271440.

5. Chemaitelly H, Ayoub H, AlMukdad S, et al. Duration of mRNA vaccine protection against SARS-CoV-2 Omicron BA.1 and BA.2 subvariants in Qatar. medRxiv 2022:2022.03.13.22272308.

6. Abu-Raddad LJ, Chemaitelly H, Ayoub HH, et al. Effect of mRNA Vaccine Boosters against SARS-CoV-2 Omicron Infection in Qatar. N Engl J Med 2022.

7. World Health Organization. COVID-19 clinical management: living guidance. Available from: <https://www.who.int/publications/i/item/WHO-2019-nCoV-clinical-2021-1>. Accessed on: May 31, 2021. 2021.

8. World Health Organization. International guidelines for certification and classification (coding) of COVID-19 as cause of death. Available from: <https://www.who.int/classifications/icd/Guidelines_Cause_of_Death_COVID-19-20200420-EN.pdf?ua=1>. Document Number: WHO/HQ/DDI/DNA/CAT. Accessed on May 31, 2021. 2021.

9. World Health Organization. COVID-19 clinical management: living guidance. Available from: <https://www.who.int/publications/i/item/WHO-2019-nCoV-clinical-2021-1>. Accessed on: May 15 2021. 2021.
