# Supplementary material for: Assessing vaccine effectiveness against severe COVID-19 disease caused by omicron variant. Report from a meeting of the World Health Organization
Source: Vaccine. 2022 Jun 9;40(26):3516–27. doi: 10.1016/j.vaccine.2022.04.069 (PMC9058052; doi:10.1016/j.vaccine.2022.04.069)
Supplement: Supplementary data 2 [file mmc2.docx]

The data in figure and Table 1 are available at <https://view-hub.org/covid-19/effectiveness-studies>.
